# New insights into human hair: SAXS, SEM, TEM and EDX for Alopecia Areata investigations

**DOI:** 10.7717/peerj.8376

**Published:** 2020-01-14

**Authors:** Adina Coroaba, Anca E. Chiriac, Liviu Sacarescu, Tudor Pinteala, Bogdan Minea, Sorin-Alexandru Ibanescu, Mihaela Pertea, Aurelian Moraru, Irina Esanu, Stelian S. Maier, Anca Chiriac, Mariana Pinteala

**Affiliations:** 1 Centre of Advanced Research in Bionanoconjugates and Biopolymers, “Petru Poni” Institute of Macromolecular Chemistry, Iasi, Iasi, Romania; 2 Department of Epidemiology, Faculty of Medicine, “Grigore T. Popa” University of Medicine and Pharmacy, Iasi, Iasi, Romania; 3 Department of Inorganic Polymers, “Petru Poni” Institute of Macromolecular Chemistry, Iasi, Iasi, Romania; 4 Faculty of Medicine, “Grigore T. Popa” University of Medicine and Pharmacy, Iasi, Iasi, Romania; 5 Department of Surgery, Faculty of Dental Medicine, “Grigore T. Popa” University of Medicine and Pharmacy, Iasi, Iasi, Romania; 6 Department of Surgery I, Faculty of Medicine, “Grigore T. Popa” University of Medicine and Pharmacy, Iasi, Iasi, Romania; 7 Clinic of Plastic and Reconstructive Microsurgery, “Sf. Spiridon” Emergency Hospital, Iasi, Iasi, Romania; 8 Romanian Academy, Bucharest, Bucharest, Romania; 9 “Dr. Iacob Czihac” Military Emergency Clinical Hospital, Iasi, Iasi, Romania; 10 Department of Internal Medicine I, “Grigore T. Popa” University of Medicine and Pharmacy, Iasi, Iasi, Romania; 11 Faculty of Textiles, Leather and Industrial Management, “Gheorghe Asachi” Technical University of Iasi, Iasi, Iasi, Romania; 12 Department of Dermatophysiology, “Apollonia” University, Iasi, Iasi, Romania; 13 Department of Dermatology, Nicolina Medical Center, Iasi, Iasi, Romania

**Keywords:** Alopecia areata, Scanning electron microscopy, Transmission electron microscopy, Energy-dispersive X-ray spectroscopy, Microbeam small angle X-ray scattering

## Abstract

**Background:**

Alopecia areata (AA) is a T-cell-mediated autoimmune disease and affects up to 2% of the population. There is a need for a more profound and rigorous understanding of the structure and composition of human hair affected by AA in order to manage this disease. The aim of this article is to understand the effects of AA on the structure and composition of human hair.

**Methods:**

Several physico-chemical investigation methods, such as Scanning Electron Microscopy (SEM), Transmission Electron Microscopy (TEM), Energy-Dispersive X-ray Spectroscopy (EDX), and microbeam Small Angle X-ray Scattering (SAXS), were used to analyze human hair samples obtained from healthy donors and patients with AA.

**Results:**

SEM revealed more severe hair surface defects for the white regrown hair (W-AA) samples. TEM showed the presence of air-like vesicles located in the endocuticle of regrown hair. Analysis of ultrathin sections of W-AA showed the existence of empty vesicles and smaller melanin granules compared to control samples. SAXS demonstrated that unaffected hair of patients with AA (B-AA) and W-AA melanin aggregates are different in their sizes and shapes compared to the control samples. EDX data showed that W-AA elemental composition was significantly different from the other sample groups. Our study showcases promising non-invasive techniques for a better and more accurate understanding of changes in the internal structure and composition of hair affected by AA.

## Introduction

Alopecia areata (AA) is a common condition that causes patchy hair loss and affects up to 2% of the population (Tobin, Fenton & Kendall, 1990a; Pratt et al., 2017; Trüeb & Dias, 2018). AA is a T-cell-mediated autoimmune disease where normal hair follicles gradually start to elicit an autoimmune response (McElwee et al., 2013; Guo et al., 2015; Lee et al., 2018). Despite seeming unimportant, hair integrity plays an important social role and its loss can become an important source of anxiety and disability (Trüeb & Dias, 2018). Hair is a filamentous material consisting mainly of 65–95% proteins, particularly coiled-coil α-keratin (Blume-Peytavi, Whiting & Trüeb, 2008; Yang, Zhang & Rheinstädter, 2014). The center contains the less structured *medulla* (which is not always present in all the hair fibers) surrounded by the more structured *cortex*. The later contains keratin, lipids and melanin, which gives the hair color. The cortex is protected by a layer of cuticles (Maugh, 1978; Blume-Peytavi, Whiting & Trüeb, 2008; Rouse & Van Dyke, 2010; Yang, Zhang & Rheinstädter, 2014). Hair follicles containing the hair bulb at their base anchor the hair into the skin. The bulb consists of living cells that provide all the nutrients for structuring and growing of hair (Tobin, Fenton & Kendall, 1990b).

AA preferentially attacks pigmented hair over white hair, and in many cases an initial regrowth as non-pigmented hair occurs (Tobin, Fenton & Kendall, 1990b; Gilhar & Kalish, 2006; Jalalat, Kelsoe & Cohen, 2014; Jia et al., 2014). Using light and electron microscopy, Tobin et al. (2002) (Tobin, Fenton & Kendall, 1990b) showed that specifically the hair bulb melanocytes were damaged in acute AA. He demonstrated that morphologic changes in the cytoplasm of damaged melanocytes result in impaired melanogenesis and the loss of the ellipsoidal shape. Epitope spreading is considered to have an important role in the broad response against melanocytes (Gilhar & Kalish, 2006; Jia et al., 2014).

Non-invasive techniques such as Scanning Electron Microscopy (SEM), Transmission Electron Microscopy (TEM), Energy-Dispersive X-ray Spectroscopy (EDX), and microbeam Small Angle X-ray Scattering (SAXS) are more frequently used for understanding and diagnosing diseases (Zhang et al., 2007; Sidhu et al., 2008; Jehle et al., 2010; Schroer et al., 2010; Chen & Kriwacki, 2018). They offer qualitative, quantitative and/or structural information regarding changes that occur in materials. SEM allows micro- and nano-scale characterization of biological structures, including hair, skin or nails (Schatten, 2013; Orime et al., 2013; Solovan et al., 2015; Coroaba et al., 2016; Turcan et al., 2016). EDX is already used in conjunction with SEM as a tool for investigating the elemental composition (concentration and distribution profile) of these structures (Kempson, Skinner & Kirkbride, 2006; Ryu et al., 2014; Coroaba et al., 2016). The main elements found in human hair are carbon, oxygen, nitrogen and sulfur, with traces of additional metals and minerals (Schroeder & Nason, 1969; Maugh, 1978; Blume-Peytavi, Whiting & Trüeb, 2008). Hair concentrations of heavy metals like arsenic, lead, cadmium and mercury, were correlated with their presence in the internal organs, blood and urine (Robbins, 2012). TEM is an essential tool to assess the homogeneity of a sample and study biological systems at near-atomic-level resolution (Wagner et al., 2007; Sato et al., 2013; Kinsler et al., 2013; Smentkowski, 2014). SAXS is a powerful method providing detailed micro- to nanoscale structural and physical information (the specific inner surface, surface to volume ratio, or lattice type and dimensions) for a variety of systems (Schnablegger & Singh, 2013). An accurate non-destructive method usually requiring a minimum of sample preparation, SAXS is routinely employed to analyze the structures of polymers (Chu & Hsiao, 2001), tissues (Fernández et al., 2002; Blanchet et al., 2015), biological macromolecules and nanocomposites in solution (Spilotros & Svergun, 2014) etc. SAXS enabled the determination of hair structure with a high spatial resolution (Kajiura et al., 2006; Yang, Zhang & Rheinstädter, 2014; Stanić et al., 2015) and it was used as a non-invasive screening method for cancer (James et al., 1999; Briki et al., 1999; James, 2001). In this context, the above techniques have the potential to turn hair analysis into a valuable tool for the investigation of certain diseases (Maugh, 1978).

**Figure 1:**
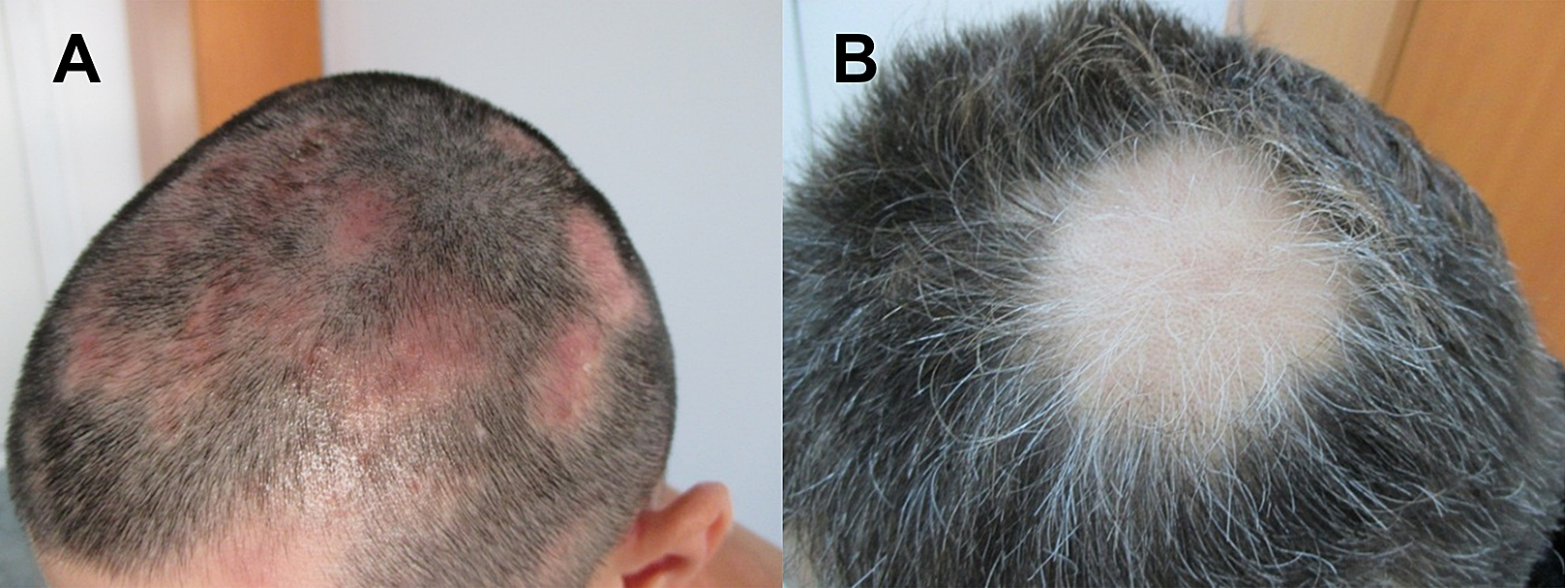
Images of patients suffering from patchy Alopecia Areata. (A) Case of patchy Alopecia areata without regrown white hair. (B) Patient with regrown white hair.

The present study is focused on the structure and composition analysis of human hair affected by AA (Fig. 1). Using SEM, EDX, TEM and SAXS, the differences between unaffected hair and white regrown hair from the same AA patients compared with white and black hair from healthy volunteers are highlighted.

## Materials and Methods

### Hair samples

Hair was collected from 25 to 60 year-old Caucasian Romanian males from Iași County. Twenty cases of patchy AA and 20 healthy donors (control hair) were investigated. For all the hair samples, five hair strands of 0.5 cm to one cm-long portions from the roots were collected. The analyses were performed on the root end. Donors with chronic diseases, those having used hair therapeutics or cosmetics (medicinal shampoos which can interfere with the results of the investigations) and those having dealt with toxic agents were excluded. Diagnosis of AA was based on clinical examination and histology performed on a fourmm punch biopsy, which excluded scarring alopecia or other hair loss causes. The patients had normal lab results including complete blood count, chemistry panel, thyroid stimulating hormone, free thyroxine, total triiodothyronine, vitamin B12, anti-thyroglobulin, anti-peroxidase and anti-parietal cell antibodies. The patients were not known to have other disorders or to have taken any medication. Two types of hair samples were collected before treatment: fine and non-pigmented regrown hair (W-AA) and unaffected hair (clinically and dermatoscopically). For this study the W-AA and G-Control samples were collected from the same scalp areas: 17 were taken from the vertex area of the scalp, one from the right temporal region and two from occipital region. The pigmented hair samples (B-AA and B-Control) samples were collected from a healthy region of the scalp.

Hair was cleaned using the Hess procedure (Hess et al., 1990): samples were placed in small dishes with distilled water containing a drop of detergent and sonicated for 5 min; hairs were then washed in distilled water, sonicated for 5 min in absolute acetone and allowed to dry. The Scientific Council of the “Petru Poni” Institute of Macromolecular Chemistry at the Romanian Academy approved the processing of biological samples collected in accordance with research law regulation EC No: 206 for this research. For all patients and volunteers, informed and written consent was obtained before initiation of any study-related procedures. The research was performed under the Helsinki ethical principles for medical research involving human subjects.

### SEM and EDX measurements

For SEM, all hair samples were mounted on aluminum alloy discs using double-sided carbon tape, and transferred to the evaporation unit, where they were uniformly coated with a 20 nm thick gold layer. The gold coating was performed on a LEICA EM SCD050 Cool sputtering device for universal SEM coating. All the specimens were examined with a Quanta 200 Scanning Electron Microscope at 30 kV in High vacuum mode. The EDX system mounted on the Quanta 200 SEM was used for element identification and quantitative analysis. The elemental analysis was performed both on the surface and on the cross section of the hair samples. The samples did not require secondary treatment or processing.

### TEM measurements

The cross sections for TEM were performed by ultramicrotomy with a Leica Ultracut UCT Ultramicrotome using a diamond knife. Hair samples were embedded in epoxy resin Epon-Araldite solution and polymerized for 36 h in a 60 °C vacuum-drying oven. The embedded hair samples were sectioned into thin cross sections using an ultramicrotome at a speed rate of four mm/s. The 50–70 nm cross sections were deposited on collodion/carbon film grids (300 mesh) and observed using a Hitachi HT7700 transmission microscope operating at 100 kV in High Contrast Mode.

### X-ray measurements of the hair samples

SAXS was used to characterize the nanometric structure of the human hair samples. The experiments were performed on a Nanostar U-Bruker system equipped with a Vantec 2000 detector (200 mm diameter) and a X-ray I µS microsource. The wavelength of the incident X-ray beam was λ = 1.54 Å (CuKα) and the beam was collimated by three pinholes. The scattered intensity I (*q*) was plotted as a function of the momentum transfer vector *q* = 4π sin θ/λ, where λ is the wavelength of the X-rays and θ is half the scattering angle. The sample-to-detector distance was 107 cm allowing measurements with *q* values between 0.008 Å^−1^ and 0.3 Å^−1^. The angular scale was calibrated by the scattering peaks of a silver behenate standard. The samples were measured under vacuum at a constant temperature of 25 °C. The background was subtracted from the original intensity profiles. SAXS-NT (Bruker integrated) and ATSAS 2.5.1 software (Konarev et al., 2003) were used for data analysis. Both control and AA samples were analyzed in identical conditions at 25 °C for 20,000 s. Samples were mounted vertically in a dedicated holder so that the X-ray beam crossed the hair perpendicularly. The same relative coordinates were used in every case. Data integration was made following the equatorial regions according to the presented diffractograms. The information obtained through SAXS came predominantly from the cortex of the hair shaft.

### 3D simulation of SAXS data

Ab initio reconstruction of protein structures was performed using computing algorithms based on the chain-like ensemble of *“dummy residues”* method. The obtained results were represented using Hyperchem rendering set up to evidence the secondary structures. Thus, both the shape of the melanin structures as well as the geometry of the primary structures (initial filaments, IF) packaging could be studied. All SAXS data were further used to get an image of the structures inside the cortex.

### Statistical analysis

Statistical analysis was performed using GraphPad Prism 8.2.1 (GraphPad software, Inc., San Diego, CA, USA). For EDX data analysis, each participant was sampled in triplicate. The three measurements were averaged and the resulting means were used to compute descriptive statistics for each group, that is, standard deviations (SD) and 95% Confidence Intervals of the means (95% CI). The groups were compared with regular two-way ANOVA. The Sidak post test was used for multiple comparisons. Statistically significant differences were considered when *p* < 0.05.

## Results

**Figure 2:**
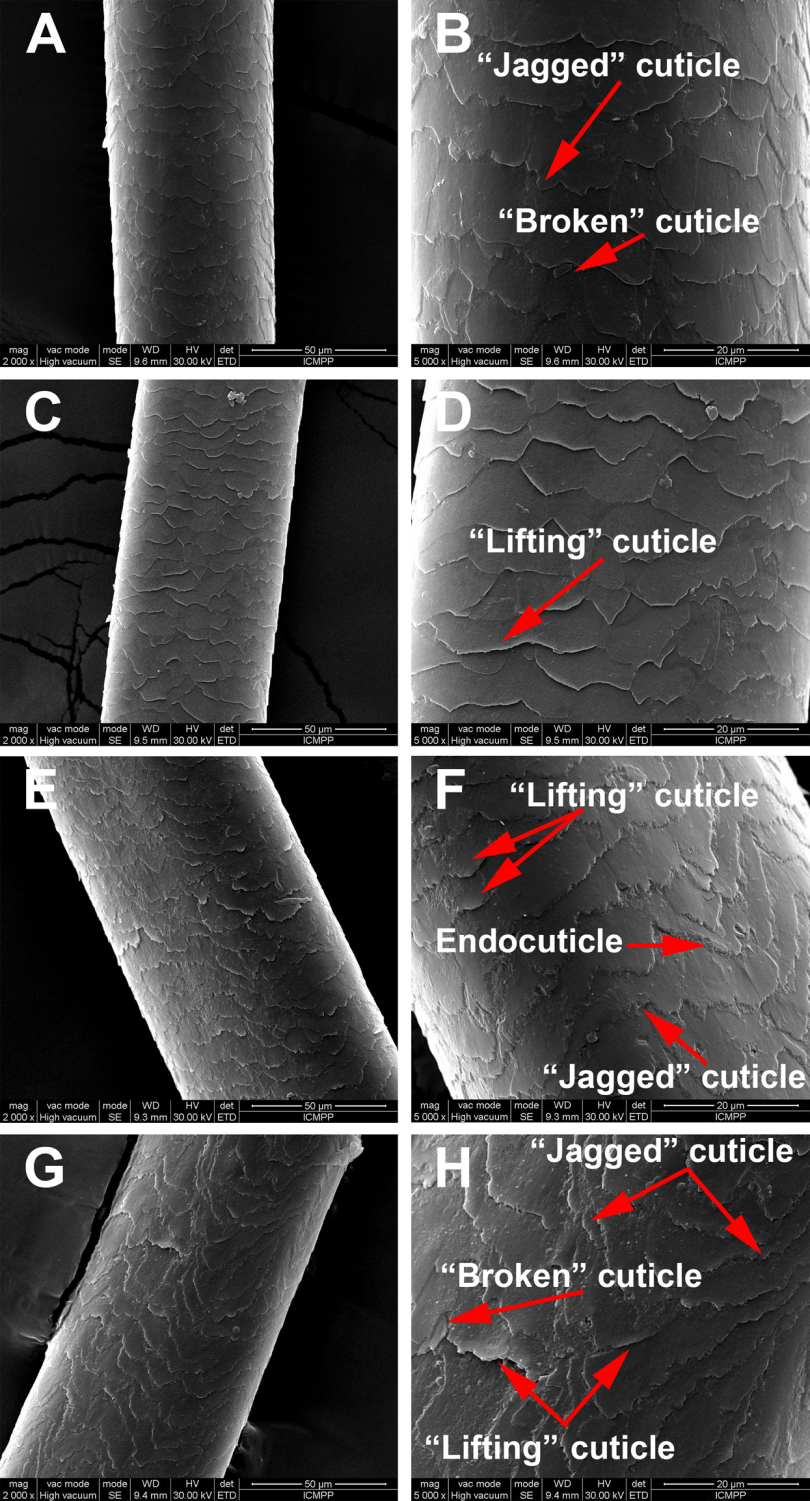
Scaning electron microscopy of hair samples. SEM images: (A and B) Black hair from healthy donor (B-Control). (C and D) Grey hair from healthy donor (G-Control). (E and F) Unaffected black hair of patient with AA (B-AA). (G and H) Non-pigmented hair of patient with AA (W-AA). Scale bars: (A), (C), (E) and (G) 50 µm; (B), (D), (F) and (H) 20 µm.

*SEM morphological investigations* revealed surface details such as hair thickness, cuticle appearance and defects due to disease or environmental factors. Figure 2 is a representation of the morphological aspects of the hair cuticles for the control and AA samples obtained through SEM analysis. The SEM micrographs show overlapping of cuticles for both the healthy donors and the AA patients. Moreover, in Fig. 2 changes to the surface morphology of the cuticles can be observed, such as “lifting cuticle layer,” “jagged cuticle” or “broken cuticle,” in both groups owing to every-day hair manipulation (washing, applying conditioner and brushing etc.) or strong wind, but these defects are more severe in hair from AA patients.

**Figure 3:**
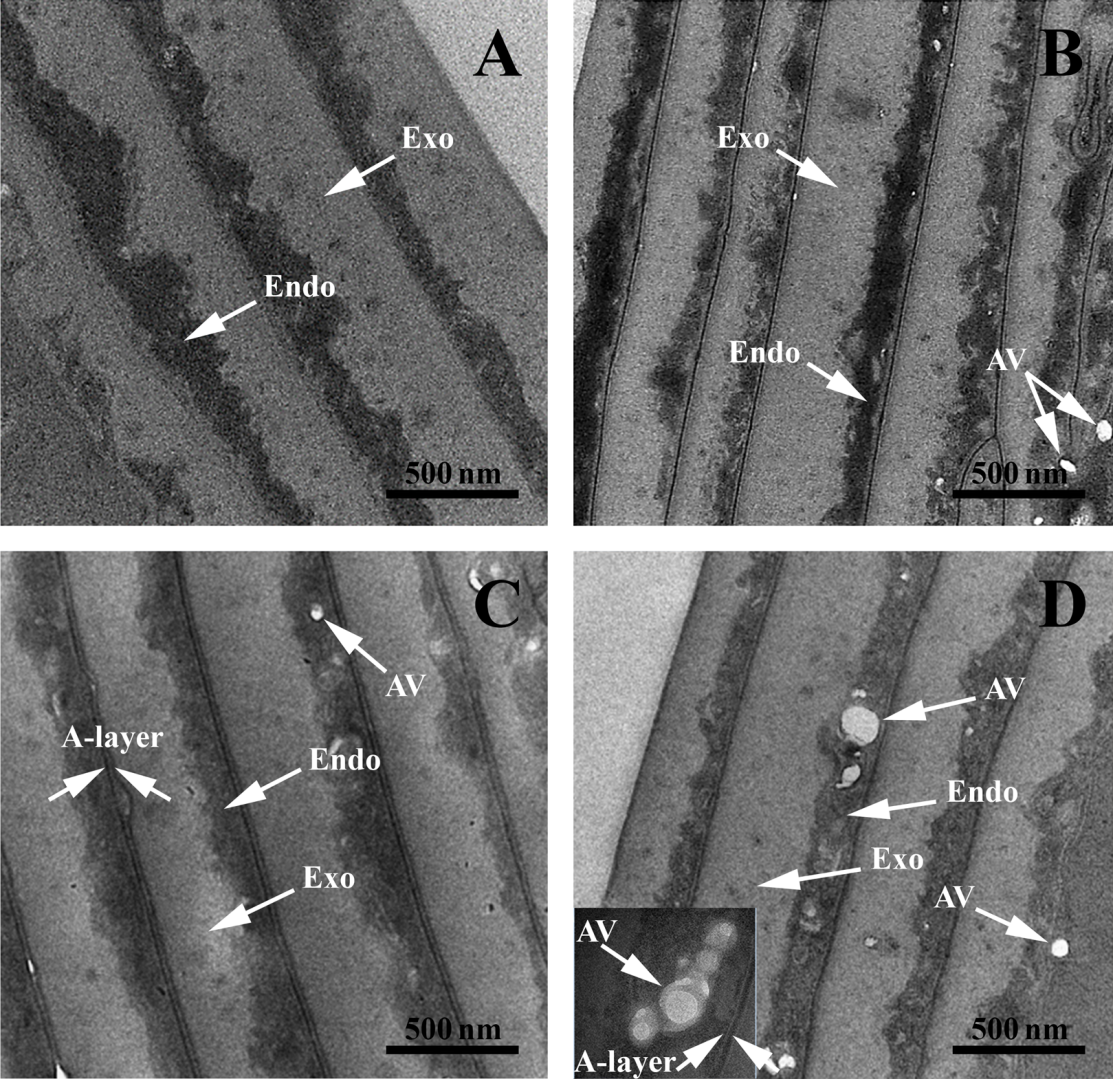
Transmission electron microscopy of hair sample cross-sections. TEM images: (A) Black hair from healthy donor (B-Control). (B) Grey hair from healthy donor (G-Control). (C) Unaffected black hair from AA patient (B-AA). (D) Non-pigmented hair from AA patient (W-AA). Scale bars: 500 nm. Exo, exocuticle; Endo, endocuticle; AV, air-like vesicles.

*TEM structural investigations* provided finer details of the internal structure and its hierarchical organization in both control and AA hair samples. The typical structures of the cuticles are highlighted in Fig. 3. All samples had well defined endocuticle and exocuticle. Additionally, gray-control (G-Control), black AA (B-AA) and white AA (W-AA) samples presented an intact A-layer with a higher electron density than the endo- and exocuticle (Popescu & Höcker, 2007; Bhushan, 2008; Sato et al., 2013). Noticeably, AA samples, and particularly W-AA (Fig. 3D), had spherical air-like vesicles (AV) located in the endocuticle.

**Figure 4:**
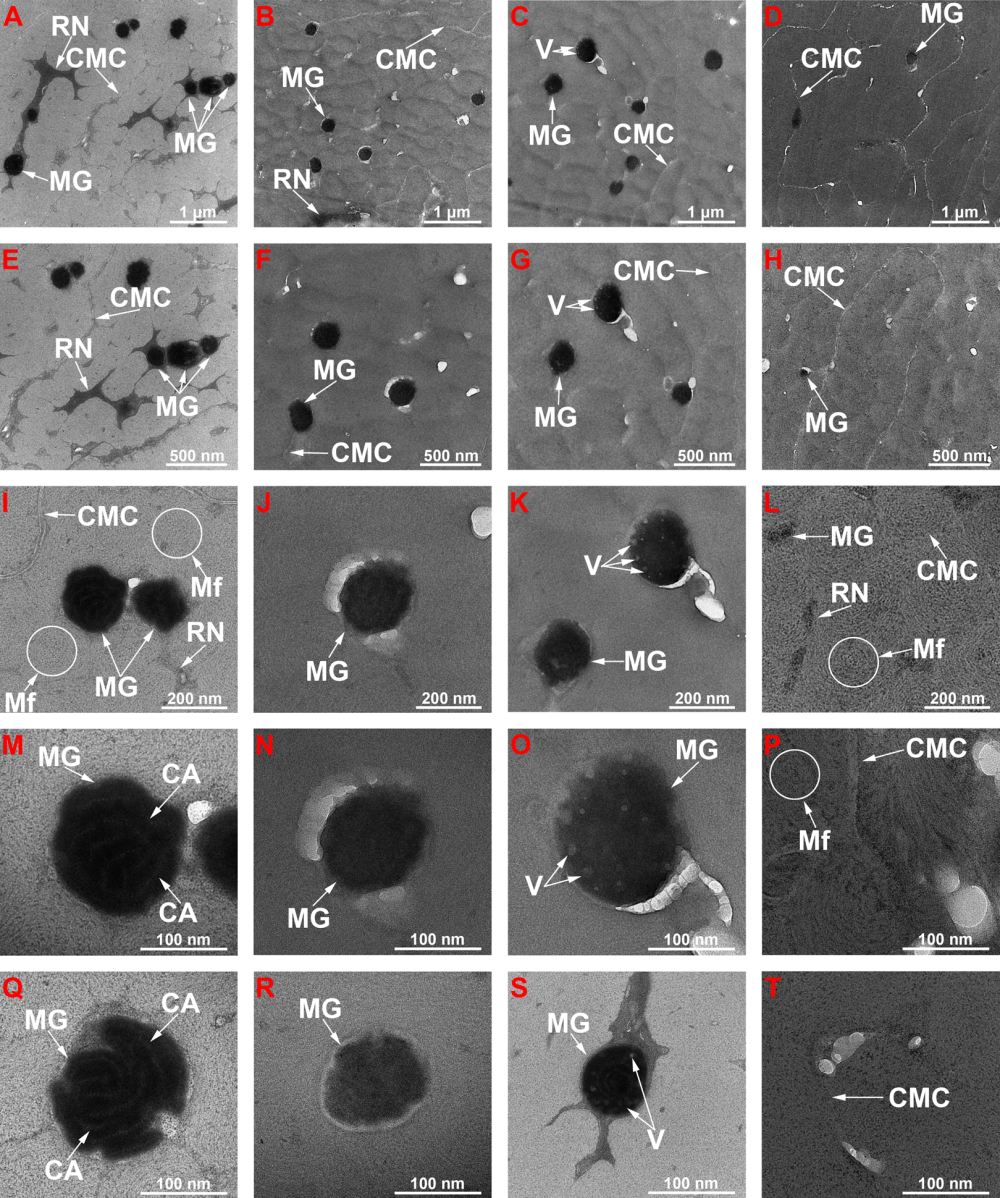
Transmission electron microscopy of ultra-thin sections of hair samples. TEM micrographs at different magnitudes for ultra-thin sections: (A, E, I, M and Q) Black hair from healthy donor (B-Control). (B, F, J, N and R) Grey hair from healthy donor (G-Control). (C, G, K, O and S) Unaffected black hair of patient with AA (B-AA). (D, H, L, P and T) Non-pigmented hair of patient with AA (W-AA). Scale bars: (A–D) one µm. (E–H) 500 nm. (I–L) 200 nm. (M–T) 100 nm. CMC, Complex membrane cells; MG, Melanine granule; RN, Remnant nucleus; Mf, Macrofibrils; CA, Crystalline aggregates; V, Vesicles.

The micrographs of the cortex areas of black-control (B-Control) (Figs. 4A, 4E, 4I, 4M and 4Q), G-Control (Figs. 4B, 4F, 4J, 4N and 4R), B-AA (Figs. 4C, 4G, 4K, 4O and 4S) and W-AA (Figs. 4D, 4H, 4L, 4P and 4T) indicate the presence of different cell types separated by a cell membrane complex (CMC) (Tobin, Fenton & Kendall, 1990a, 1990b; Tobin et al., 2002). In W-AA, melanin granules (MGs) were small and very rare or absent and more empty vesicles were noticed. TEM of B-Control (Figs. 4M and 4Q) revealed the presence of smaller crystalline aggregates inside the melanin granule, while the B-AA samples (Figs. 4O and 4S) had MGs with internal vesicles, which were more prominent in B-AA hair.

**Figure 5:**
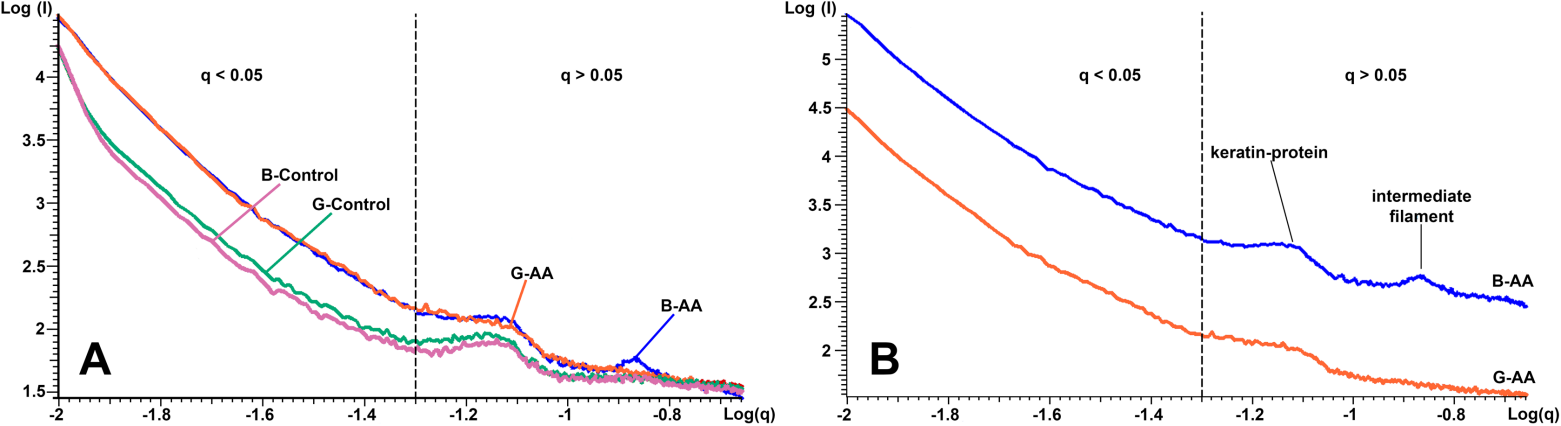
SAXS diffraction data of hair samples. SAXS diffraction data: (A) Graph of B-Control, G-Control, B-AA and W-AA samples. (B) Offset of SAXS diffractogram for B-AA and W-AA samples. The two areas of interest (*q* < 0.05 Å^−1^ and *q* > 0.05 Å^−1^) are delimited by a dashed line.

*The SAXS* results presented in Fig. 5 shows in logarithmic scale the diffraction data of the cross-section of control and AA hair samples. “The scattering curves” for all analyzed samples showed two areas of interest, delimited by the dashed line (Figs. 5A and 5B). The first area was located at *q* < 0.05 Å^−1^ and the second at *q* > 0.05 Å^−1^ and contained two more or less obvious correlation peaks. The results were analyzed in relation to the two mentioned areas. On a wide range of vector values, scattering intensity was considerably lower for control samples. A profile of this kind suggests the presence of scatterers of significantly larger dimensions in the control cortex area. The decrease in intensity was linear on the value range *q* < 0.05 Å^−1^, and the slope, −α ≈ 3. Both features (linearity and slope value) indicated the existence of scattered fractal geometry.

The second are in the *q* > 0.05 Å^−1^ value range the scattering curve showed two correlation peaks at *q* = 0.14 Å^−1^ and *q* = 0.07 Å^−1^. They expressed structural features of IFs. They had characteristics that differentiated B-AA and W-AA samples (Fig. 5B, the curves have offset for a better understanding). For B-AA, the two peaks were strongly contoured, while for W-AA only the peak from *q* = 0.07 Å^−1^ was clearly visible. With this exception, the scattering curves corresponding to the two samples overlap perfectly in this *q* range.

**Figure 6:**
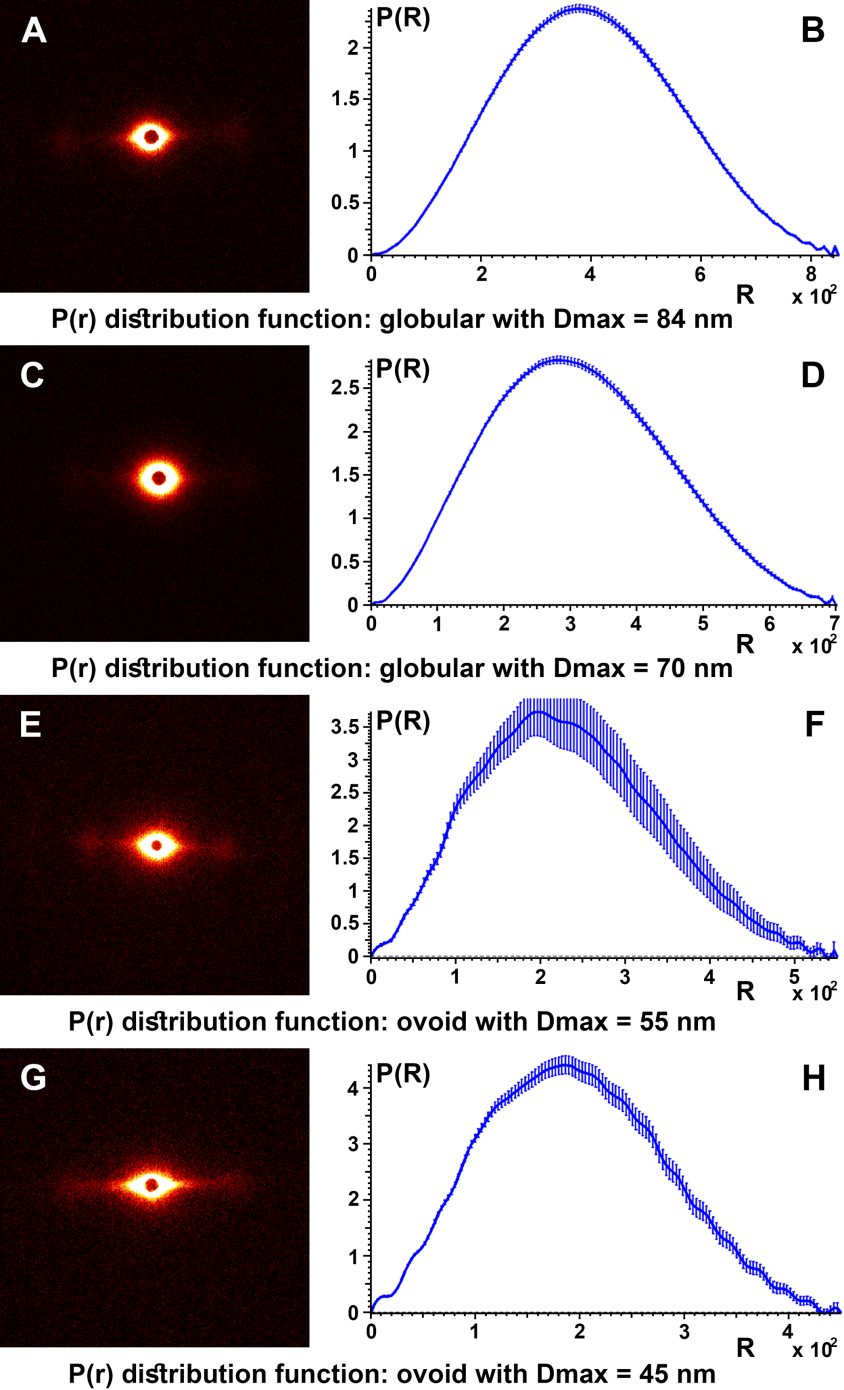
SAXS diffractogram and P(r) distribution function. SAXS diffractogram and P(r) distribution function: (A and B) Black hair from healthy donor (B-Control). (C and D) Grey hair from healthy donor (G-Control). (E and F) Unaffected black hair of patient with AA (B-AA). (G and H) Non-pigmented hair of patient with AA (W-AA).

To obtain details about the packing structures of the samples, experimental SAXS data were used to calculate the so-called pair distance distribution function P(r) by the inverse Fourier transform (Fig. 6). Typically, the experimental points located at the beginning of the scattering curve are affected by the interference phenomena between scatters. These were not taken into account for the obvious aim of obtaining a better representation for the shape factor and a smooth convergence to zero at the point corresponding to the maximum dimension of the structure (D_max_). Therefore, for all control samples the P(r) function had a “bell-shape” characteristic of a spherical structure. The maximum dimension of the structures is obtained at the point where the function decreases to “0.”

**Figure 7:**
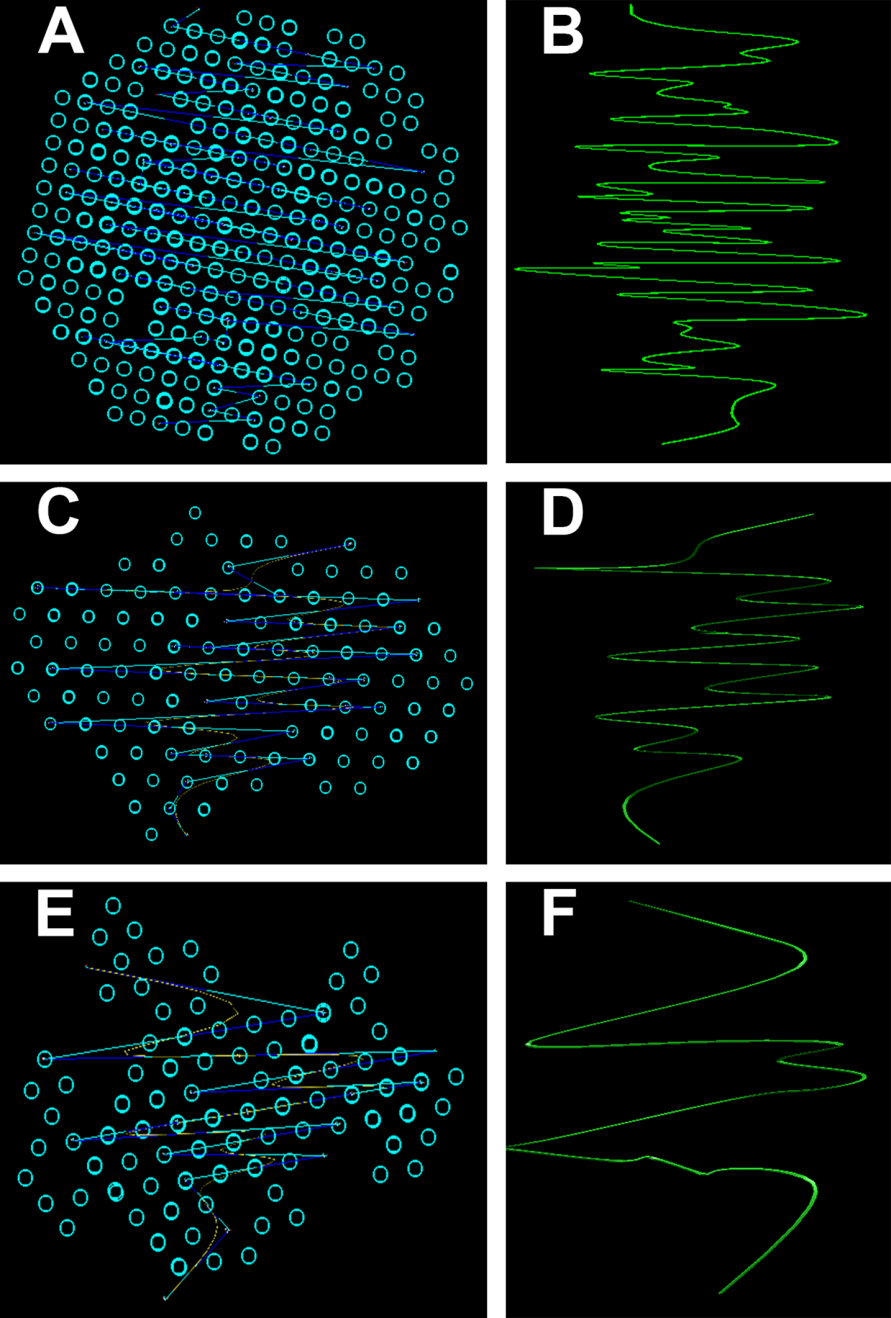
Ab initio reconstruction of the (protein) structures. Ab initio reconstruction of the (protein) structures: (A and B) Black hair from healthy donor (B-Control). (C and D) Unaffected black hair of patient with AA (B-AA). (E and F) Non-pigmented hair of patient with AA (W-AA). The blue circles represent dummy atoms used to generate the continuous phase representing the melanin texture. Inside the continuous phase, the method highlighted a second phase as a (helicoidal line) that could be assigned to the primary structure (initial filaments). The graphical interface was used to represent the biphasic system of melanin with the help of two panels: the left (A), (C) and (E) shows the general graphical image of the biphasic melanin system, the continuous phase (blue circles) having inside IF (line) as the second phase; the right (B), (D) and (F) is a graphical representation highlighting only the aspect of the second phase, IF (green lines).

*3D Simulation of SAXS data* was the next step in our analysis. This involved the ab initio reconstruction of the (protein) structures using the computing algorithms based on the chain-like ensemble of *dummy residues* method (Fig. 7). This method it is used to represent multiphase molecular systems, that is, particles. For this purpose, the particle’s multiphase model is generated using densely packed “dummy atoms.” Such model is characterized by a configuration vector that assigns to each atom a certain phase. This structure (model) is then subjected to a simulated relaxation to obtain a configuration that best fits the experimental data. The results were graphically represented to highlight the secondary structures. Thus, both the shape of the keratin structures as well as the geometry of the primary structures IF packaging could be studied. All the information resulted from SAXS analysis were further used to get an image of the structures inside the cortex of the studied hair filaments. The blue circles represent dummy atoms used to generate the continuous phase representing the melanin texture. Inside the continuous phase, the method highlighted a second phase as a (helicoidal line) that could be assigned to the primary structure IF. The graphical interface was used to represent the biphasic system of melanin with the help of two panels: the left panel shows the general graphical image of the biphasic melanin system, the continuous phase (blue circles) having inside IF (line) as the second phase; the right panel is a graphical representation highlighting only the aspect of the second phase, IF (green lines).

**Figure 8:**
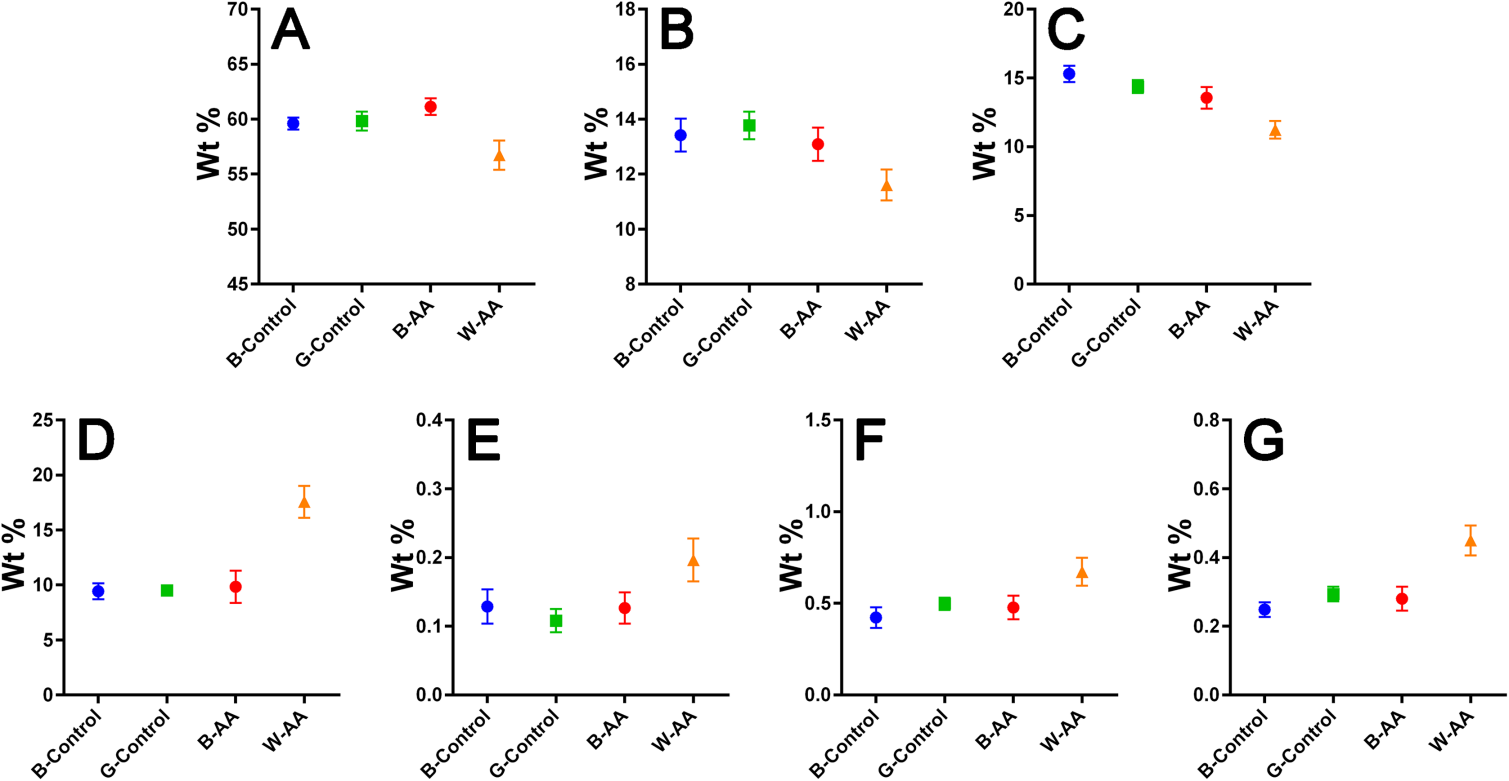
EDX elemental composition. Means with 95% CIs: (A) Carbon. (B) Nitrogen. (C) Oxygen. (D) Sulfur. (E) Iron. (F) Copper. (G) Zinc.

*Elemental composition—EDX investigations* (Table 1) showed the elemental compositions of hair from the healthy volunteers and unaffected hair from AA patients to be similar, without statistically significant differences (Table 2). The white regrown hair from the affected areas (WAA), however, was significantly different from the other three categories of hair. Figure 8 shows reduced levels of C, N and O, and increased levels of particularly S, but also Fe, Cu and Zn in the WAA hair.

**Table 1:** Average concentration for elemental composition obtained from EDX analysis (Wt%).

Element	Statistic	B-Control	G-Control	B-AA	W-AA
C	Mean ± SD 95% CI	59.60 ± 1.15 59.07−60.14	59.81 ± 1.85 58.95–60.68	61.14 ± 1.64 60.37–61.90	56.72 ± 2.86 55.38–58.06
N	Mean ± SD 95% CI	13.42 ± 1.28 12.82–14.02	13.78 ± 1.08 13.27–14.28	13.09 ± 1.30 12.48–13.69	11.61 ± 1.19 11.05–12.16
O	Mean ± SD 95% CI	15.30 ± 1.26 14.71–15.89	14.37 ± 1.01 13.90–14.84	13.56 ± 1.67 12.77–14.34	11.23 ± 1.35 10.60–11.86
S	Mean ± SD 95% CI	9.43 ± 1.54 8.71–10.16	9.51 ± 0.74 9.17–9.86	9.84 ± 3.13 8.37–11.30	17.56 ± 3.11 16.11–19.02
Fe	Mean ± SD 95% CI	0.13 ± 0.05 0.10–0.15	0.11 ± 0.04 0.09–0.13	0.13 ± 0.05 0.10–0.15	0.20 ± 0.07 0.17–0.23
Cu	Mean ± SD 95% CI	0.42 ± 0.12 0.37–0.48	0.50 ± 0.07 0.46–0.53	0.48 ± 0.14 0.41–0.54	0.67 ± 0.16 0.60–0.75
Zn	Mean ± SD 95% CI	0.25 ± 0.05 0.23–0.27	0.29 ± 0.05 0.27–0.32	0.28 ± 0.07 0.25–0.31	0.45 ± 0.09 0.41–0.49

**Table 2:** Two-way ANOVA of EDX elemental composition: *p* values adjusted for multiple comparison.

Element	B-Control vs. G-Control	B-Control vs. B-AA	B-AA vs. W-AA	G-Control vs. W-AA
C	0.94	0.06	<0.0001	<0.0001
N	0.28	0.56	0.0015	<0.0001
O	0.14	0.06	<0.0001	<0.0001
S	0.99	1.00	<0.0001	<0.0001
Fe	0.66	0.54	0.0004	0.0008
Cu	0.06	0.16	<0.0001	<0.0001
Zn	0.68	0.99	<0.0001	<0.0001

## Discussion

The cuticles observed from *SEM morphological investigations* consist of stacked layers of keratin and are responsible for the visual appearance and the tactile properties of hair (Blume-Peytavi, Whiting & Trüeb, 2008; Jolles & Zahn, 2011; Robbins, 2012; Maeda et al., 2018). Based on cystine content, the cuticles are classified into endocuticle, A-layer, exocuticle, and epicuticle (Blume-Peytavi, Whiting & Trüeb, 2008; Bhushan, 2008; Jolles & Zahn, 2011).

The changes to the surface morphology of the cuticles observed in hair from AA patients (i.e., “lifting cuticle layer,” “jagged cuticle” or “broken cuticle”) could be the consequence of changes in chemical composition, that is, a lower content in cystine and, therefore, less disulfide bridges in the A-layer (Robbins, 2012), and more cystine degradation products. Because the attribute of the cystine is to confer stiffness to the hair, its lowering content can lead to a pronounced degradation on the hair surface (Pollitt & Stonier, 1971; Cruz et al., 2016).

*TEM structural investigations* revealed spherical AV located in the endocuticle. These vesicles can cause a lower hair density, which can explain its fragility (Kim et al., 2010). The micrographs of the cortex areas highlighteddifferent cell types separated by a CMC. Cortical cells consist mostly of bundles of keratin intermediate filaments called macrofibrils (Mf) and MGs as spherical black structures. Typically, keratin fibers with acidic groups are associated with those containing basic residual groups forming coiled-coils (Yang, Zhang & Rheinstädter, 2014; Stanić et al., 2015). These dimers are also antiparallel and associated into tetrameric spirals. The tetramers interconnect head to tail and give rise to protofilaments. Tetramers (protofilaments) form so-called intermediate filaments (IFs) (Popescu & Höcker, 2007). MF, MGs, remnant nucleus and CMC are the main structures that could be observed.

*SAXS measurements* suggested that, on average, in the AA samples (B-AA, W-AA) melanin structures had smaller dimensions since the scattering intensity was higher than in the control samples. In addition, in control samples, the appearance of the scattering curves did not change, irrespective of the color of the hair fiber. Their profiles were almost identical to those obtained for AA sample; the small existing differences suggest a spherical shape for the MG in control samples and an ovoid shape in the AA samples. Dense packing of IFs produced two visible equatorial peaks at *q* = 0.14 Å^−1^ and *q* = 0.07 Å^−1^. These signals were derived from X-ray scattering reported in the literature (Kreplak et al., 2001; Stanić et al., 2015) and were characteristic of spiraled keratin proteins. Thus, the signal at *q* = 0.07 Å^−1^ (*d* = 90 Å) represents the distance between IFs in human hair and *q* = 0.14 Å^−1^ (*d* = 45 Å) corresponds to the internal structure of IFs resulting from spiraled protein (two or four spirals)—tetrameric oligomers (protofibrils). Therefore, in W-AA the IFs are structurally affected in the keratinic protein packing area (the number of coiled coils).

The pair distance distribution function P(r), for all control samples, irrespective of the color of the hair, leads to a maximum size of the globular aggregates in the range D_max_ = 67–84 nm and for the target samples (B-AA and W-AA) the estimated maximum dimensions in the range D_max_ = 45–55 nm. In this case the variation of the function showed small oscillations and the profile was slightly altered suggesting an ovoid shape.

From the *3D Simulation of the SAXS data* it was observed that in all the cases the IF structures showed a specific coiled-coils helicoidal pattern which was different from the references. Moreover, there was a remarkable difference concerning the structure consistency even between the samples. The frequency of the spiral structure elements was very high in the case of control samples, especially for the dark hair filaments (B-Control). Also, even though their frequency was lower for the G-Control filaments, the difference was still considerable compared to the samples of affected hair. Both B-AA and W-AA contained a much smaller number of spiraled stacked packages than the control samples. Also, B-AA contained a slightly larger number of elements within the spiral package than W-AA where this structure was barely visible.

In B-Control (Fig. 7), IF spirals were well represented with dense helical elements. The organized structures were globular and had relatively large dimensions (70–80 nm). In AA samples, the organized structures were strongly deformed and smaller, reaching about 50 nm. Initial filament packaging density also decreased dramatically, especially for W-AA (45 nm). Thus, the AA samples presented strong differences relative to the control samples. These differences were found at the structural level in the cortex. Here, the biological material is organized on two measurable levels, both being accessible in the SAXS field. A first domain refers to the shape and size of the primary structures, that is, polydispersed melanogenic aggregates. The second one refers to the intimate structure of the IFs (keratin aggregates). This type of organization was found in all analyzed samples regardless of their origin. The essential differences between AA and control samples resulted from the packaging of filaments (secondary structures) and from the shape/dimension of the organized structures. The number of filaments in W-AA was much reduced compared to control samples (see the packing density) while in B-AA, the number of filaments was slightly higher, but still much smaller than in the control samples. Therefore, the modifications within the affected hair samples were certainly the result of a health condition that affected hair formation and growth.

*The elemental composition resulted from EDX investigation* revealed reduced levels of carbon (C), nitrogen (N) and especially oxygen (O) in W-AA samples. This could be explained in part by the increased percentage of sulfur, but also by the lack of melanin (Hallégot, Peteranderl & Lechene, 2004), a substance that could be described as a polymer of oxidized tyrosine (Solano, 2014). In the case of white hair from healthy volunteers, the change in color is not due entirely to the reduction of melanin synthesis, which happens gradually, over time, but also to changes in the geometry and size of melanosomes in the hair. In other words, the white is not just chemical, but also a physical color (Popescu & Höcker, 2007; Tobin, 2008), hence the similarities with the healthy pigmented hair. In AA, however, although the melanocytes are not entirely destroyed, the immune attack appears to bring their proliferation, differentiation and melanin production to a complete stop (Gilhar, Etzioni & Paus, 2012), which would explain the differences in C, N and O composition between the two types of white hairs.

The contribution of multiple biological processes can be invoked to explain the increased levels of sulfur (S), iron (Fe), copper (Cu) and zinc (Zn) in W-AA samples. Firstly, melanin itself reduces the proliferation of keratinocytes and induces their differentiation towards corneocytes (Tobin, 2008). In its absence, the proliferation continues, which requires significant amounts of Zn and Cu (Kil, Kim & Kim, 2013; Ogawa, Kawamura & Shimada, 2016). Copper is also important for keratinization (Whang et al., 2004). Secondly, the auto immune attack in AA induces a marked oxidative stress in hair producing structures (Bakry et al., 2014). Zinc, Cu and Fe are important components of antioxidant and anti-inflammatory mechanisms (Kuvibidila et al., 2013; Ogawa, Kawamura & Shimada, 2016). Furthermore, metallothioneins, which are cysteine-rich Zn/Cu-binding proteins, are upregulated in keratinocytes as a response to oxidative stress (Ogawa et al., 2018). They are one possible explanation for the increased level of sulfur. This consumption of zinc for both keratinocyte proliferation and fighting oxidative stress could explain the reduced Zn serum levels reported for patients with prolonged AA (Jin et al., 2017). The role of Fe in hair formation is not yet clear (Ruiz-Tagle et al., 2018). Iron is, however, the cofactor of ribonucleotide reductase, the limiting enzyme for DNA synthesis, an essential process in actively proliferating keratinocytes (Jin et al., 2017). This is one possible factor contributing to the elevated Fe levels of W-AA hair.

## Conclusions

Here, hair samples from patients with patchy AA and healthy donors were investigated using SEM, TEM, SAXS and EDX and compared with samples from healthy donors. Their analysis revealed morphological and structural differences in both the surface and cross-sectional areas of hair. The samples from patients suffering from AA presented significant differences in chemical composition, presenting less melanin but also in its distribution, melanin aggregates being less frequent and smaller. One limitation of our study was the non-quantitative nature of the SEM and TEM analyses, which were intended as a visual support in interpreting SAXS and EDX data.

Overall this article demonstrates that human hair analysis offers possibilities for deep investigation of several other maladies or disabilities. The non-invasive techniques used in our study could provide exciting opportunities in practical medical applications as diagnostic tools and/or detection of degradation mechanism induced by a specific disease or compound.

## Supplemental Information

10.7717/peerj.8376/supp-1 Supplemental Information 1 Raw Data: Elemental concentrations obtained from EDX analysis.

## Additional Information and Declarations

### Data Availability

The following information was supplied regarding data availability: The raw data is available in a Supplemental File.
